# Group Membership Affects Spontaneous Mental Representation: Failure to Represent the Out-Group in a Joint Action Task

**DOI:** 10.1371/journal.pone.0079178

**Published:** 2013-11-20

**Authors:** Jennifer Susan McClung, Ines Jentzsch, Stephen David Reicher

**Affiliations:** 1 Institute of Biology, University of Neuchâtel, Neuchâtel, Switzerland; 2 School of Psychology and Neuroscience, University of St Andrews, St Andrews, Scotland, United Kingdom; University College London, United Kingdom

## Abstract

Predicting others’ actions is crucial to successful social interaction. Previous research on joint action, based on a reaction-time paradigm called the Joint Simon Task, suggests that successful joint action stems from the simultaneous representation of the self with the other. Performance on this task provides a read-out of the degree of intrusion from a partner that participants experience from acting jointly compared to acting alone, which in turn is a measure of the degree to which participants mentally represent their co-actors during the task. To investigate the role of perceived group membership in this type of joint action and its influence on the representation of others, we first subjected participants to a minimal group paradigm while manipulating differences in social competition. We then asked participants to do the Joint Simon Task in pairs with an in-group or out-group member. Only participants who acted with an “in-group” partner on the joint task showed altered reaction times compared to when acting alone, presumably a change caused by the simultaneous and automatic representation of their in-group partner. In contrast, participants who acted with an out-group partner were unaffected in their reactions when doing the joint task, showing no evidence of representation of their out-group partner. This effect was present in both the high-competition and low-competition conditions, indicating that the differential effects of group membership on representation during joint action were driven by perceived group membership and independent of the effects of social competition. We concluded that participants failed to represent out-group members as socially relevant agents not based on any personality or situational characteristics, but in reaction only to their status as “other”. In this way group membership appears to affect cognition on a very immediate and subconscious level.

## Introduction

Group membership has well-documented and striking effects on human social behaviour. From the quotidian to the more rare, the range of effects of group membership on behaviour is vast. For instance, parliamentary discussion and debate take place along party lines drawn up by ministers’ ideological group membership, and, more significantly, wars may last over years when two groups cannot reconcile their differences. In experimental settings, even membership in artificially formed groups of strangers based on arbitrary categories can have a profound impact on behaviour. In a classic study, Tajfel and colleagues [1] experimentally formed such ‘minimal’ groups based on an arbitrary and randomly assigned criterion (supposed ability to estimate the numbers of dots presented on a screen) and showed that participants preferentially rewarded members of their own group and punished members of the ‘out-group’. Although these minimal groups were in fact formed randomly, participants’ rewarding and punishing behaviour was nevertheless significantly affected by the perceived group membership of others [1]. In a more socially relevant setting, people were more likely to help an injured in-group member in need of physical assistance while ignoring out-group members in the same state [2]. Group membership also has documented effects on more basic behaviours, such as physical proximity maintenance. People have been shown to approach in-group members more and conversely to establish a larger inter-individual distance with perceived out-group members [3].

Group membership also affects certain aspects of cognition. For instance, once categorised as an out-group member, people are more easily dehumanised than perceived in-group members and consequently they are attributed fewer secondary emotions (considered to be uniquely human emotions) such as sorrow, admiration, and contempt [4]. Out-group members are also attributed fewer human values and traits in favour of more animalistic qualities [5] and less intelligence [6].

While we know from this research that group membership has a powerful influence on behaviour and that some of the cognitive processes that proceed from categorisation based on group membership are well described, less is known about the most fundamental cognitive processes involved in the generation and maintenance of such group biases. The most basic cognitive processes that facilitate and maintain differential categorisation, and the often detrimental responses to others based on perceived group membership, are less well understood. In this study we attempt to address this deficiency by investigating whether group membership affects one of the most basic, subconscious aspects of social cognition, the automatic representation of others during a joint action task.

In order to jointly act with another person, one is required to understand that person’s current behaviour and to be able to predict their future behaviour [7]. One of the classic methods used to study joint action is a slight alteration of what was originally called the ‘Simon Task’ [8]. In the Simon Task, a participant is required to press one of two buttons in response to one feature of a compound stimulus, such as the colour of an arrow (e.g. red = left key; blue = right key), while ignoring another feature, such as its spatial orientation (e.g. pointing right or left). The ‘Simon Effect’ occurs when participants react faster to stimuli in which the (irrelevant) spatial dimension of the stimulus corresponds to the response location, and slower to non-corresponding stimuli. For example, if a participant is instructed to respond to red with the left key and to blue with the right key, she will respond quicker to red arrows pointing left and to blue arrows pointing right and slower to the opposite combinations. Interestingly, if participants are only given one half of the task, for example to only respond to red stimuli (by pressing the left key) and to ignore blue stimuli, then the Simon Effect disappears; the irrelevant spatial dimension no longer interferes with response time when participants do only half of the task.

**Figure 1:**
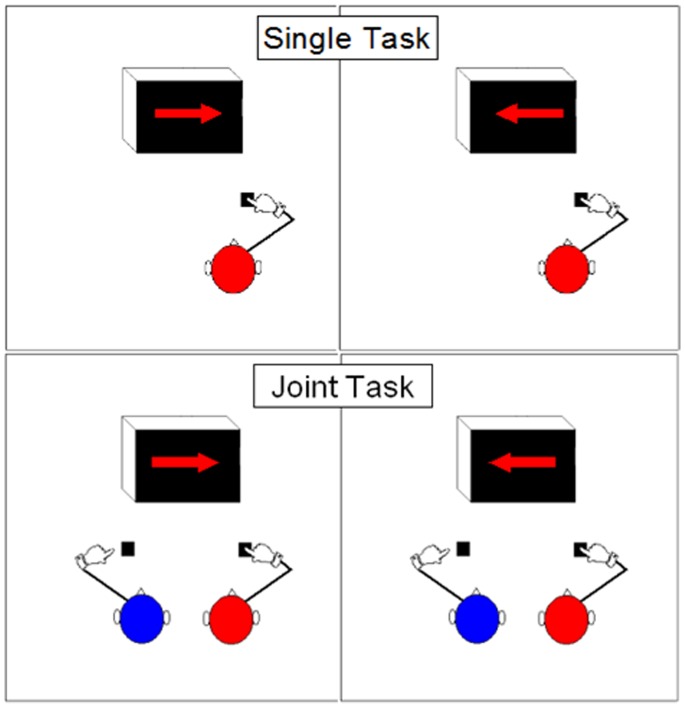
Schematic of the experimental tasks, showing compatible (left panel) and incompatible trials (right panel), for the Single Task (top) and the Joint Task (bottom) from the perspective of the red participant. In the top Single Task boxes no Simon Effect occurs (compatible and incompatible responses are equally fast), but in the bottom ‘Joint Task’ boxes there is a Social Simon Effect (a slowing of reactions to incompatible stimuli and a speeding of reactions to compatible stimuli).

The alteration of the Simon Task to explore joint action in a social context entails adding another participant to form the ‘Joint Simon Task’. Here, two participants jointly act by each doing one half of the task simultaneously (e.g. participant A is tasked with responding to red stimuli; participant B with responding to blue stimuli; see fig. 1). Importantly, although being asked to do exactly the same as in the previous restricted half-version of the Simon Task (which usually produces no Simon Effect), in the joint task the Simon Effect reappears. Researchers have dubbed this the ‘Social Simon Effect’ [9]. The originally observed interference of the irrelevant spatial dimensions recurs even though the demands on each participant in the joint task are the same as in the individual half-task version.

Although certain conditions (such as schizophrenia) may prevent self-other integration and lead to a lack of representation during joint action [10], in general, adults consistently represent their partner during this task as evidenced by the presence of a Social Simon Effect [11]. In recent years, however, some complexities in relation to this paradigm have been reported. Notably, the critical condition for representation of the other (as evidenced by the presence of a Social Simon Effect) is not their mere presence. What seems to be critical is whether the other is active and intentional - or rather, perceived to be active and intentional. Thus, on the one hand, participants do not represent partners who are present but inactive [12], [13]. On the other hand, they do represent others whom they believe intend to cooperate on the task, even if these others are physically absent and no auditory or visual feedback is received from them [12], [14]–[16]. From these findings, it has been reasoned that the Social Simon Effect is a function of joint action such that people represent not only their own but also their partner’s actions simultaneously and automatically (for example [12], [17]–[20]). It has further been argued that this type of mental ‘co-representation’ is a necessary precondition for successful joint action (such as cooperation) [21].

These studies open up the path to examining how co-representation is a function of the social relations between people, and a small body of work has begun to address this issue. Thus, evidence suggests that people preferentially represent those they perceive in a socially positive way, showing an increased Social Simon Effect compared to when people interact with a partner who is negatively perceived [22]. Other work has looked at the effect of group membership on representation. We are aware of two studies that have addressed the effects of group membership on the Social Simon Effect, but the results of these studies have been contradictory. One study used racial differences, examining how people perform when interacting with a hand which is either of their own skin colour or that of a different racial group. This study showed that the Social Simon effect occurred when participants interacted with a racial in-group but not with a racial out-group [19]. The other study used a minimal group categorisation and found no effect of group membership on the Social Simon Effect [23].

There are a number of ways of explaining this inconsistency. For instance, it could be argued based on the results of the first study that the Social Simon effect is not a function of group membership per se (that is, whether the other is in-group or out-group) but rather of the specific norms, values, and histories associated with these racial groups. Thus, for instance, because of a tradition of racial antagonism participants might assume that the racial ‘other’ will not cooperate in, and hence be irrelevant to, the task at hand, with the result that there is no Social Simon Effect in the out-group condition. Alternatively, it could be argued that the Social Simon Effect may be a function of group membership itself, but that the minimal group manipulation used in the second study was too weak and ephemeral to impact on the subsequent task: participants simply stopped thinking of themselves (and others) in terms of their group membership. Clearly, then, any investigation of whether group membership itself impacts on representation of the other must use a manipulation which is robust enough to structure how people perceive their co-actor.

Another, and more nuanced, explanation has to do with the fact that the group manipulation may be expected to work at two different levels. That is, as well as impacting on a representational level, group membership may affect levels of motivation. People inherently compete with out-group members [24] even when concrete resources are not at stake [25]. Their aim is to gain comparative social status by proving their superiority over the out-group [26]–[28]. Thus one would expect people to strive harder when interacting with out-group members as opposed to with in-group members.

Applying this general argument to the Social Simon paradigm, the increased effort resulting from social competition may be expected to speed up all response times when interacting with out-group members. This could lead to a ceiling effect which would mask the Social Simon Effect. In other words, the motivational effects of the group membership manipulation may obscure its representational effects. This needs to be addressed in order to clarify whether representation of another person is indeed moderated by their perceived group membership.

We address these various concerns in the present study. First, we employed a more robust operationalisation of the minimal group paradigm [1]. That is, we divided people into categories (‘dot over-estimators’ and ‘dot under-estimators’) that have no real world significance in terms of prejudice or interaction with each other. However, we ensured that participants were reminded of their category membership throughout the study, including when they carried out the Joint Simon task.

Second, in order to parcel out the effect of motivational differences between encounters with in-group and out-group members, we introduced an explicit manipulation of social competition. That is, in one condition (competition) we stressed that we were comparing the two groups (over-estimators and under-estimators) to determine who would perform better and therefore participants should try to do well. In the other condition (no-competition) we avoided all mention of competition or comparison between the groups and told participants they could relax and get comfortable. Thus, although we might expect overall differences in performance between these two competition conditions because of the different levels of motivation they induce, the important point is that within each we would expect the motivational dimension to be constant across interactions with in-group vs out-group members. This allowed us to address the impact of group membership on co-representation without the motivational confound.

Our predictions were that participants would only represent another’s actions if the partner was perceived as an in-group member. That is, that the ‘Social Simon Effect’ would only occur between individuals who were jointly acting as members of the same group. We also predicted that group membership should be the only factor to influence the Social Simon Effect, regardless of the level of social competition, although our social competition manipulation may affect the overall reaction time.

## Method

### Ethics Statement

Approval to conduct this study was granted by the University Teaching and Research Ethics Committee (UTREC) at the University of St Andrews prior to running participants, permit number PS5019.

### Participants

Sixty-four students at the University of St Andrews (age range: 17–20 years) participated. In order to avoid gender effects, or the possibility that gender identity would confound group identity, all participants were female. All were tested in pairs (32 pairs) in a single session lasting approximately 30 minutes. All participants were naive to the experimental hypothesis, gave informed consent, were fully debriefed at the end of the study and received course credit for participation. Participants were randomly assigned to one of four experimental conditions: out-group vs. in-group X competition vs. no-competition. Four additional pairs of participants were tested but excluded from analysis: in three of these pairs, participants incorrectly used two hands instead of one to respond in the single task, and in the other pair one participant had joint problems caused by severe arthritis which impeded her reaction abilities.

### Materials and Procedure

The experiment was conducted in the EEG Lab in the Psychology Department at the University of St Andrews. Before the experiment it was confirmed that participants did not know each other. Participants arrived in pairs at the lab and were given instructions before any chatting could take place. They were given information forms describing the experiment and then asked to complete a written consent form.

We first used a minimal group paradigm to randomly allocate pairs of participants to either an out-group or in-group condition. As mentioned above, minimal group paradigms are used to vary participants’ group memberships relative to each other using the most minimal criteria possible [1]. Ostensibly, participants were put into categories on the basis of a trivial criterion - in this case, whether they over-estimated or under-estimated the number of dots in a series of patterns - although in reality they were randomly assigned to be over-estimators or under-estimators. Importantly, by using a minimal group paradigm, we could ensure that the ‘groups’ in our experiment had no significant or real-world meaning to participants in terms of prejudice, no history of interaction, or other implicit associations.

At the beginning of the experiment, participants were told that the study was aimed at investigating the relationship between cognitive style and reaction times. This was intended as a rationale for the manipulation of group membership using the minimal group paradigm. To that end they were told that the experimenter would first assess their cognitive style and then ask them to complete a computerized reaction task.

**Figure 2:**
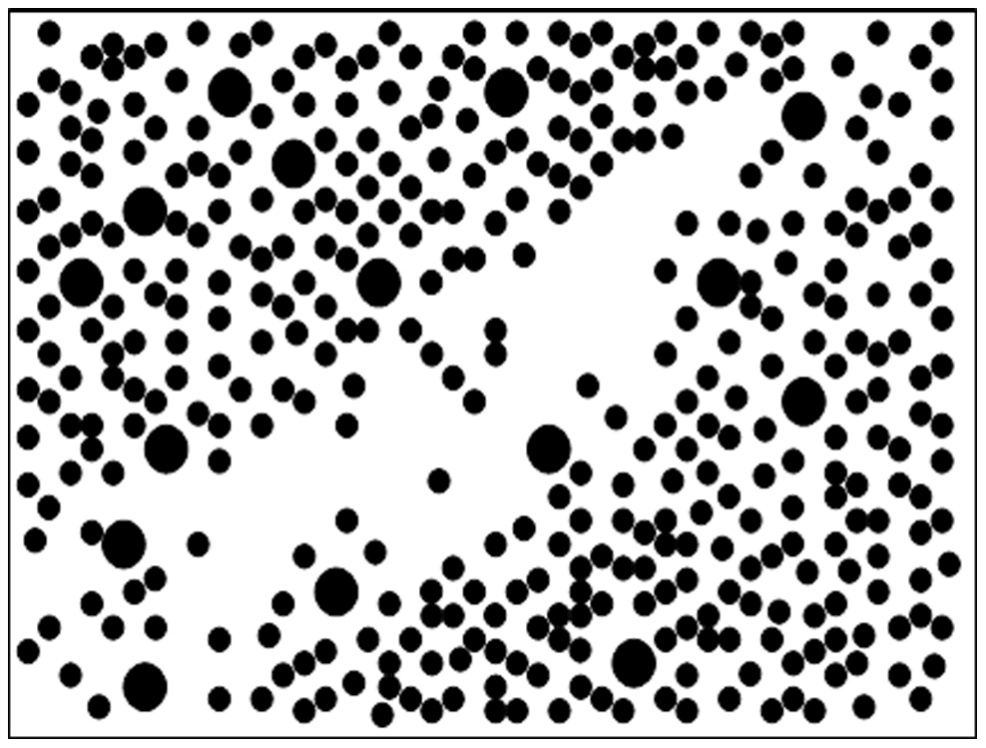
Representative illustration of a ‘Dot Estimation Task’ picture.

Their cognitive style, they were told, would be assessed using a test called the ‘Dot Estimation Task’ (DET), which was in reality the minimal group paradigm used to categorize participants into out-group and in-group conditions (adapted from [29]). Participants were given some theoretical background of the DET in order to give credibility to the cover story. They were told that their performance on the DET would be indicative of their cognitive style in general since estimation abilities correlated strongly with other aspects of cognition. The DET itself involved estimating the amount of dots present on three consecutive pictures (made using Microsoft Power Point, see fig. 2 for an example below). Dot pictures were presented for 3 seconds each using Microsoft Power Point and a projector.

Participants were asked to do the task alone in order to ‘get a clear and true read-out’ of their cognitive style. In actual fact this request was designed to keep participants from discussing their answers and thereby realizing that there was no actual correlation between estimates and assigned category. The experimenter then made a brief show of calculating the average of their estimations, and then randomly assigned each participant to be either an over-estimator or an under-estimator. To ensure that these categories remained salient throughout the experiment, participants were then asked to wear a badge with their cognitive style on it, ostensibly so that the experimenter ‘would not forget who is who for the purposes of data analysis’. After random allocation, 16 of the pairs remaining in the study formed the out-group condition and the other 16 pairs constituted the in-group condition.

Next, the social competition manipulation was introduced in order to standardise high and low levels of motivation across the group conditions: this formed the ‘competition’ and ‘no competition’ conditions respectively. In the ‘competition’ condition participants were made aware that they would be compared with the ‘other’ group – whether that was their partner in the task or participants doing the task at another time. Specifically, participants were told that their performance in the joint action computer task was correlated with cognitive abilities and general intelligence and as such we would use the task to determine whether over-estimators or under-estimators were ‘better’ on the reaction time task. They were then asked to perform as well so that we could obtain an accurate measure of each group’s capabilities.

In the ‘no-competition’ condition we minimised the inherent social competition participants may have felt by avoiding all reference to the potential comparison or competition between the different identities. Participants were instead told that they would be given extensive practice on the computer task before the ‘actual experiment’ (which never came), and that they could use the practice sessions to ‘relax and just get comfortable’ with the task. No mention of participants’ group-based performance or comparison was made.

Participants then carried out the Joint Simon Task, which consisted of responding to stimuli presented on a CRT monitor using Experimental Run Time Software (ERTS). The stimuli consisted of red or blue coloured arrows 35 mm long and 4 mm wide, presented centrally on black background, pointing either to the left or the right. Responses were recorded using ERTS keypads. All participants performed the task alone (single task) and together with a partner (joint task), with task order counter-balanced across participants to exclude any effect of task order on performance. In the single condition, an empty chair remained beside each participant. In the joint task, participants sat side-by-side in front of the monitor (see fig. 1 for a schematic of the task).

Participants were instructed to respond to arrows of one colour only and to withhold the response to arrows of the other colour. In the joint condition the other participant was assigned the other colour, whereas in the single condition no one was assigned to the other colour. The four stimulus alternatives (red or blue arrows pointing to the left or right) were presented randomly and with equal probability. For each task, two experimental blocks (8 practice and 64 experimental trials each) were presented, separated by short rests. Stimuli were presented until response or a maximum of 1500 ms, followed by a 1000 ms blank interval. After each block all participants received feedback on their accuracy but not speed.

At the end of each joint action task, participants were then asked to complete a short questionnaire which consisted of the following sections: 1) general interest in the partner’s mental states, 2) similarity/closeness that they felt to their partner, 3) the importance of their identity as a student to them, and 4) the degree to which they felt social competitive pressure. Each section consisted of 6 items, 3 negative and 3 positive, requiring likert-scale responses from 1 (‘strongly disagree’) to 7 (‘strongly agree’). See Questionnaire S1 for full questionnaire. Participants were fully debriefed at the end of their session and given course credit.

## Results

### Data Analysis

All trials with incorrect, too fast (<100 ms), or too slow responses (>1000 ms) were discarded from reaction time analysis (<1% of overall trials). For each task (joint and single), reaction time means for participant pairs were subjected to a 2 X 2 X 2 repeated-measures ANOVA with the within-subject variable Compatibility (compatibility vs. incompatibility) and the between-subject variables Group Status (in-group vs. out-group) and Competition (competition vs. no-competition). We did not analyse error rates as accuracy was very high (98% correct). Again, the key prediction was that participants would represent their partners less when asked to jointly acting with out-group members than with in-group members. The Social Simon Effect, we predicted, should occur only between in-group members. We also predicted that this effect should occur in the in-group condition regardless of the level of social competition because the level of social competition in itself should not affect whether a partner is perceived as an intentional co-actor. Therefore, the level of social competition should affect overall reaction time (producing faster reaction times in the ‘competition’ condition), but should not alter the Social Simon Effect itself.

### Overall Interactions

Because of our a priori hypothesis of a Social Simon Effect present only in the joint condition we focus below on the joint and single tasks separately and performed ANOVA’s on each. For the sake of clarity, we also include here the single and joint task in one omnibus ANOVA, with within subject variables task (joint vs single) and compatibility (compatible vs incompatible trials) and the between subject variable group membership (inter-group vs intra-group conditions). The key interaction (task × compatibility × group membership) was almost significant (F (1,28) = 4.13, p = 0.052). Further, there was no main effect of group overall (F(1,28) = 1.08, p>.3) but there was an overall main effect of competition (F(1,28) = 4.36, p<.05), such that, as expected, responses were faster in the competition conditions.

However, as suggested by several authors [30], there can be significant interaction effects within a model even if the omnibus test is not significant. Individual ANOVA’s are therefore warranted especially when, as in the current experiment, the researchers have a clear a priori hypothesis. In addition, some researchers have cast doubt on the sensitivity of omnibus ANOVAs and their ability to detect interactions [26], [30]. Wahlsten [31], for example, suggested that the sample size required for the detection of interaction effects is seven to nine times larger than that needed to detect main effects. We therefore focus our analysis on the joint and single tasks separately, as we had clear a priori predictions based on previous research findings, namely that the Social Simon effect would be present only in the joint task, and within the joint task only in the in-group conditions.

### Joint Task

Within the joint task, a main effect of competition was found, in that reaction times were faster in the competition (274.7 ms) than the no-competition condition (288.8 ms, F(1, 28) = 5.23, p<.05, η_p_^2^ = 0.16), showing that competition had a general effect.

There was no main effect of group on overall response times (in-group mean = 276.2 ms, out-group mean = 287.4 ms, F(1, 28) = 3.2, p<.08, η_p_^2^ = 0.05), as expected. More importantly, a main effect of compatibility was found: participants were faster in compatible (trials in which the spatial orientation of the stimulus and response location were the same) than incompatible trials (in which the spatial orientation of the stimulus and response location were different), (F(1, 28) = 6.50, p<.05, η_p_^2^ = 0.19), providing evidence of the usual ‘Social Simon Effect’.

**Figure 3:**
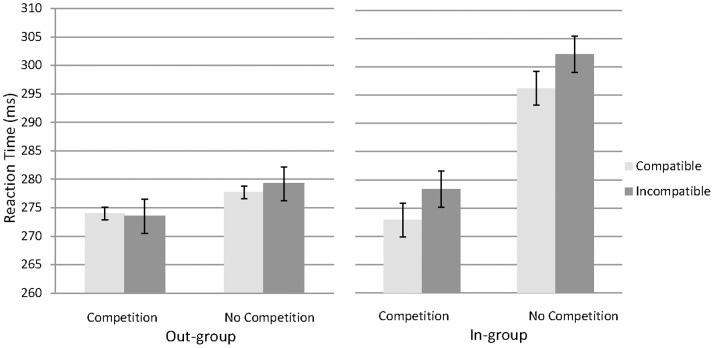
Participant reaction times (means ±SE) on the Joint Task in the Out-group (left panel) and In-group (right panel) conditions as a function of competition and compatibility.

Most critically, though, there was an interaction between group and compatibility conditions (F(1, 28) = 4.47, p<.05, η_p_^2^ = 0.14; fig. 3). The interaction was due to a significant difference between compatible (284.6 ms) and incompatible trials (290.3 ms) in the in-group condition (F(1, 28) = 7.76, p<.05, η_p_^2^ = 0.36), but not the out-group condition (compatible: 275.9 ms vs. incompatible: 276.4 ms; F(1, 28) = 0.2, p>.10; fig. 3). As predicted, the ‘Social Simon Effect’ was present in the in-group but not out-group condition. Importantly, there was no interaction between group, compatibility, and competition (F(1,28) = .29, p>.60, see fig. 3). That is, the selective presence of the Social Simon Effect in the in-group condition was not modulated by competition. Lastly, there was no interaction between competition and compatibility (F(1,28) = .30, p>.5).

### Single Task

**Figure 4:**
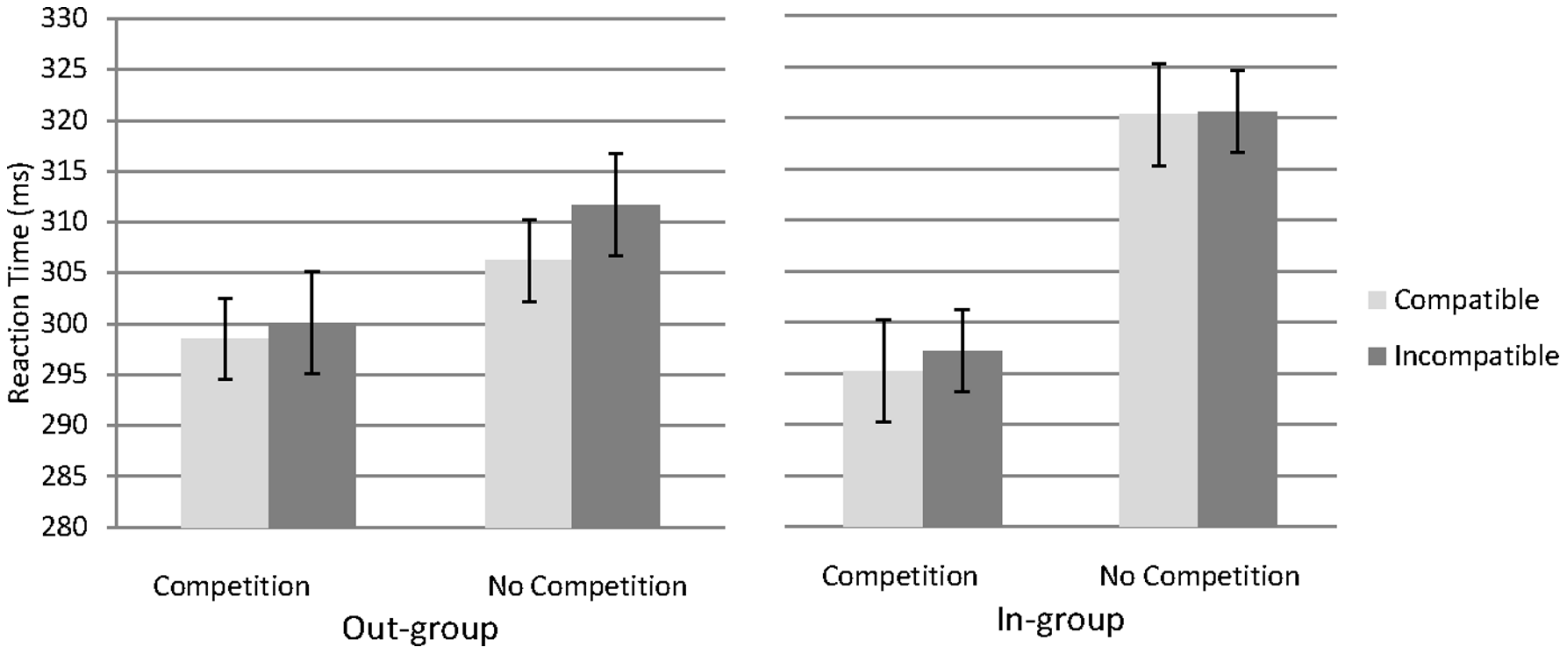
Participant reaction times (means ±SE) on the Single Task in the Out-group (left panel) and In-group (right panel) conditions, as a function of competition and compatibility.

Using the same ANOVA as described above in the joint task, we found a marginal main effect of social competition, with reaction times slightly faster in the competition condition (298 ms) than no-competition condition (315 ms), F(1, 28) = 3.17, p<.07, η_p_^2^ = 0.10. No main effect of group (in-group mean = 308.3 ms, out-group mean = 304.1 ms) was found in the single task, F(1, 28) = .22, p>.6, η_p_^2^ = 0.06. No main effect of compatibility (compatibility mean = 305.0 ms, incompatibility mean = 307.4 ms) was found in the single task, F(1, 28) = 2.10, p>.10, η_p_^2^ = 0.03, showing that the Simon Effect was indeed social and only occurred in the joint task. There was also no interaction in the single task condition between group membership and compatibility, F(1, 28) = .54, p>.10, η_p_^2^ = 0.01, or between group membership, compatibility, and competition, (F(1, 28) = .74, p>.10, η_p_^2^ = 0.10, see fig. 4).

### Questionnaire

The questionnaire consisted of four subscales, each with six items, three positive items and three negative items (see Questionnaire S1 for full questionnaire). The four subscales were: 1) mental states: general interest in the partner’s mental states, 2) similarity/closeness: degree of similarity and closeness they felt to their partner, 3) student identity: the importance of their identity as a student to them, and 4) social competition: the degree to which they felt competitive pressure with their partner. After reverse scoring the appropriate items, the internal consistency of each subscale was measured by calculating Cronbach’s alpha for each subscale. The mental states subscale, which sought to gauge participants’ interest in their partner’s mental states, had an acceptable internal reliability (α = .74). The similarity/closeness subscale, which gauged participants’ sense of closeness and similarity to their partners, also had an acceptable internal reliability (α = .76). The student identity subscale gauged the importance of the student identity to participants and was found to have low reliability (α = .55), which did not increase with removal of any one item or any combination of items. Given the low internal reliability, the student identity subscale was subsequently dropped. Lastly, the competition subscale gauged participants’ sense of competition during the computer task and was found to be highly reliable (α = .90). See table 1 below for a comparison of means.

**Table 1:** Means of the three reliable subscales as a function of group membership (in the left two columns) and competition (in two right columns).

	In-group (mean)	Out-group (mean)	Competition (mean)	No-competition (mean)
Interest in Partner’s Mental States	4.6	4.0	4.4	4.2
Similarity/Closeness	4.8	4.3	4.6	4.5
Competition	4.1	4.2	4.3	4.0

Interestingly, on the mental states subscale which measured general interest in the partner’s mental states, participants in the in-group condition reported significantly more interest (mean = 4.6) than did participants in the out-group condition (mean = 4.0), F(1, 28) = 4.7, p<.05. There was no main effect of competition on this subscale (no competition mean = 4.2, competition mean = 4.4; F(1, 28) = .67, p>.40), and no interaction between competition and group condition (F(1, 28) = .02, p>.90).

The similarity/closeness subscale assessed participants’ feelings of connection to and commonality with the partner (based on the Leach scale [32]). Participants in the in-group condition reported increased feelings of similarity and closeness to their partners (mean = 4.8) relative to participants in the out-group condition (mean = 4.3; F(1, 28) = 4.2, p<.05), but again there was no main effect of competition on this subscale (competition mean = 4.6, no competition mean = 4.5; F(1, 28) = .67, p>.40) and no interaction between competition and group condition (F(1, 28) = .23, p>.6).

Against expectations, on the competition subscale, participants in the competition condition did not report a significantly increased sense of social competition (mean = 4.3) relative to participants in the no-competition condition (mean = 4.0; F(1, 28) = 1.1, p>.40). There was also no main effect of group membership in reported feelings of competition (out-group mean = 4.2, in-group mean = 4.1; F(1, 28) = .18. p>.70), nor an interaction between competition and group membership on this subscale (F(1, 28) = .50, p>.4).

## Discussion

In this study we investigated whether people represented others during joint action as a function of their group membership. Using the Joint Simon Task [33] we replicated the well-established finding of a Social Simon Effect, in that participants doing the individual half of the task experienced no interference from the irrelevant spatial dimension, but they were significantly affected if asked to carry out the same task jointly with a partner. This reflects the extra computational demands in mentally representing the partner’s actions simultaneously with one’s own. In our study, the Social Simon Effect was not uniformly present but was instead significantly affected by basic, minimal group categorisation. That is, the Social Simon Effect was only present when participants believed they were interacting with a fellow in-group member and not with an out-group member. Results are thus consistent with the interpretation that participants failed to represent out-group members in the same way they did the more socially relevant in-groupers during these interactions.

As we noted in the introduction, previous conclusions made about the effects of group relationships on the Social Simon Effect have been in conflict with each other. One previous study that found an effect of group membership on the Social Simon Effect did not use minimal groups [19], meaning the effects could have been attributed to the relationship between the specific racial groups involved rather than simple group membership per se [34]–[37]. In another study, where the groups involved were minimal, no impact of group membership on the Social Simon Effect was found [23].

Why the discrepancy between this previous study using minimal groups and our own? Why did the previous study fail to find a difference in the Social Simon Effect between people doing the task with in-group members compared to out-group members? We have suggested two factors that may be involved: first, a weak minimal group paradigm, and second, the masking effects of resource competition. Firstly, the operationalisation of the minimal group divide used in the previous study may have simply not been strong enough: it may have failed to make the categories salient to participants while they completed the task. In effect, they stopped thinking about whether their partner was in-group or out-group. Accordingly, we changed the procedure so as to provide a constant visual cue to group membership throughout the study. Although the previous study provided no manipulation checks, our own questionnaire data clearly indicate that our group divide was relevant to people.

On the one hand, participants interacting with a presumed in-group partner reported more interest in their partners’ mental states than those interacting with a presumed out-group partner. This self-reported interest covered notions such as a partner’s ‘thoughts’, ‘beliefs’, ‘needs’, ‘intentions’, ‘knowledge’, and ‘aspirations’. Crucially, they had no opportunity to talk with their partner before completing the questionnaire, which suggests that this differential interest is a result of the group membership manipulation only. On the other hand, participants in the in-group condition experienced more feelings of similarity and closeness to their partners than did participants in the out-group condition. Both of these findings are indicative of the fact that we were successful in prompting participants to view their partner as an in-group member in the in-group condition and as an out-group member in the out-group condition.

The second factor that we discussed in the introduction concerned the potential role of motivation due to inherent social competition and its potential to mask differences between the representation of in-group and out-group members. The lack of a Social Simon Effect found in the previous study using minimal groups [23] could have resulted from the masking effect of resource competition, as operationalized by a differential rewards scheme. In that study, resource competition would have been active in addition to the natural increase in social competition that attends any interaction with an out-group member [27], [28], [38] and the two could have confounded each other. Accordingly, we explicitly manipulated the level of social competition (to make the competition vs. no competition conditions) with the aim of ensuring that, within each of these conditions, the level of social competition (and resulting motivation and effort) would be constant across the in-group and out-group conditions. We therefore expected that, while there would be an overall effect of social competition (in that responses would be faster in the competition condition due to the greater effort expended) it would not interact with participants’ representations of each other. This is exactly what we found, in that there were no interactions between social competition and group membership or the Social Simon Effect. This suggests that we have been successful both in controlling the motivational effects caused by the social competition in the minimal group paradigm and also that the selective presence of the Social Simon Effect in in-group conditions cannot be attributed to motivational processes.

Nonetheless, it is necessary to raise one caveat. That is, in this instance the questionnaire data do not provide further supportive evidence. Participants in the competition condition reported no more increased sense of urgency, motivation, or desire to beat their partner than did participants in the no-competition condition. This might suggest that our competition manipulation was ineffective and may not have had the intended effect. However, there are a number of reasons not to take the questionnaire responses on the ‘competition’ items at face value. First, there are social desirability issues [39]. That is, people do not wish to be seen as overly competitive and wanting to defeat their rivals, and this would be expected to decrease their willingness to report high levels of competition [40]. This type of bias against reporting socially undesirable phenomena is common when participants respond via self-report measures such as questionnaires [40].

Second, there are issues of comparison level. That is, people may assess their levels of competitiveness in relation to what they believe to be the norm, which in this case are the norms set up by our manipulation. That is, people who may usually consider themselves only slightly competitive may instead rate themselves as highly competitive when they compare themselves to the norm set up during their trial of being ‘relaxed and comfortable’. Conversely, those who are actually more competitive may instead rate themselves as less competitive when they compare themselves to the norm set up in our manipulation by asking them to ‘do their best due to provide a comparison with the out-group’.

In sum, we are more inclined to take seriously the behavioural data which indicate that our social competition manipulation was effective, that it motivated people to expend more effort in the competitive conditions, and that this was expressed in faster responses on all trials. Nonetheless, we would repeat our original caveat: these claims clearly need to be read in the context of our discrepant questionnaire findings.

One other qualification should be borne in mind. This study was conducted on an all-female sample, which makes generalized conclusions speculative. This, however, was planned to avoid the effects that gender has on categorisation. From a young age people spontaneously use gender to categorise social partners as in-group or out-group to themselves [41]. More specifically, gender differences can interact with and confound categorisation [42], [43], especially categorisation based on such minimal conditions as dot estimation. What is more, most of the classic studies using minimal groups have used males [1], [3], [25], [28] and as such indicate that minimal group divisions have a robust effect on the other gender. There is no reason to expect that there would be gender differences in the specific effects we have examined in this study. Nonetheless, future studies may consider using wider and more diverse samples of participants.

There is some controversy regarding the nature of the representations involved in the Joint Simon Task. A handful of studies have shown Social Simon Effects dependent upon spatial arrangement [9], [44] and/or on timing or rhythm [45], [46] with these researchers concluding that the partner in a joint Simon Task is merely used as a spatial reference point. While this ‘referential coding’ interpretation of the Social Simon Effect is possible, it does conflict with other evidence showing (a) that the Social Simon Effect occurs even when partners are imagined humans and therefore not present in the space next to a participant [16] and (b) that there is no Social Simon Effect when participants do the task alone but with an inactive person sitting next to and providing a spatial reference [12]. However, even if, for the sake of argument, we assume the referential coding interpretation of the Simon Effect to be true, and assume that people do code their partner as a mere spatial reference during the task, then, if anything, it would render the present results more striking. This would suggest that out-group members do not even warrant use as a spatial referent. In other words, we can overlook the very existence in space of an out-group member sitting right beside us. Whether or not one accepts this, and whatever position one takes concerning the level on which the other is represented, the bottom line is that these results suggest that people fail to represent out-group members as socially relevant co-actors.

We conclude with three brief comments on the wider significance of our findings. First, it is important to note that people are not consciously aware of responding differently to in-group and out-group members. When debriefed at the end of the experiment, every participant reported that during the experiment they had not considered the fact that their co-actor was in the same or a different group, and that they had all responded to the best of their ability, seemingly unaware and regardless of the condition they were in. This is not to say that they were unaware of which group they and their partner were in. Instead, they were unaware of how group membership impacted their thoughts and actions. This clearly renders the effect more pervasive and harder to control.

Second, the results presented here are in line with results from studies using electroencephalographic (EEG) and transcranial magnetic stimulation (TMS) measurements of group-based perception. Gutsell and Inzlicht used EEG measurements to show that participants demonstrate perception-action coupling (activity in the motor cortex from merely observing an action being carried out) only when observing an in-group member perform an action and not when observing an out-group member perform the same action [47]. Using TMS measures Avenanti and colleagues have shown that participants also react empathetically (by showing neural responses to perceived pain) only when watching in-group members in pain and not when observing the same in out-group members [48]. Our results contribute to these demonstrations of the fundamental impact group membership has on cognition by suggesting that it extends also to the spontaneous mental representation of a partner during joint action.

Third, and more speculatively, we would expect such fundamental effects to have consequences for more complex social cognition. For instance, without even representing another person that one is interacting with, it would be difficult to feel what they feel or to view them as having human-like mental states. In other words, the lack of representation of out-group members as shown in this study may form the basis of many behaviours from bias against the out-group to active dehumanization. This may further help us to better understand the categorical limits on our ability to cooperate [49], or to empathize [49], [50].

In conclusion, using a minimal group paradigm – the most stripped down manipulation of social group context – the current study suggests that during social interactions people differentially represent those they have categorized as ‘other’: indeed, they behave as if they were doing the task in isolation. When the other is an out-group member, it seems, we can be psychologically alone even when physically together.

## Supporting Information

Questionnaire S1 **Questionnaire given to participants at the end of the computer-based Joint Simon Task.**
(DOCX)
