# Supplementary material for: Group Membership Affects Spontaneous Mental Representation: Failure to Represent the Out-Group in a Joint Action Task
Source: PLoS One. 2013 Nov 20;8(11):e79178. doi: 10.1371/journal.pone.0079178 (PMC3835841; doi:10.1371/journal.pone.0079178)
Supplement: Questionnaire S1 — Questionnaire given to participants at the end of the computer-based Joint Simon Task. (DOCX) [file pone.0079178.s001.docx]

**Supporting Information**

End of Experiment Questionnaire: Study 3

Questionnaire – Cognitive Styles & Stimuli Processing Experiment

Please indicate how much you agree with each statement.

Key:

7 = Stongly Agree

6 = Agree

5 = Slightly Agree

4 = Neutral

3 = Slightly Disagree

2 = Disagree

1 = Strongly Disagree

**1) GENERAL INTEREST**

1) I am interested in what the other participant is thinking.

1 2 3 4 5 6 7

Strongly Disagree Neutral Strongly Agree

2) I don’t really care about what kind of beliefs the other participant has.

1 2 3 4 5 6 7

Strongly Disagree Neutral Strongly Agree

3) I feel little desire to know about the other participant’s personal needs.

1 2 3 4 5 6 7

Strongly Disagree Neutral Strongly Agree

4) I am curious about the other participant’s intentions.

1 2 3 4 5 6 7

Strongly Disagree Neutral Strongly Agree

5) Broadly speaking, I’d like to share in the other participant’s knowledge.

1 2 3 4 5 6 7

Strongly Disagree Neutral Strongly Agree

6) I am not overly concerned with the other participant’s aspirations.

1 2 3 4 5 6 7

Strongly Disagree Neutral Strongly Agree

**2) SIMILARITY / CLOSENESS**

7) I think I am similar to the other participant.

1 2 3 4 5 6 7

Strongly Disagree Neutral Strongly Agree

8) The other participant and I quite probably have many things in common.

1 2 3 4 5 6 7

Strongly Disagree Neutral Strongly Agree

9) I don’t feel a sense of ‘being connected’ with the other participant.

1 2 3 4 5 6 7

Strongly Disagree Neutral Strongly Agree

10) I don’t feel I would be able to form a bond with the other participant.

1 2 3 4 5 6 7

Strongly Disagree Neutral Strongly Agree

11) The other participant and I may be more alike than we know.

1 2 3 4 5 6 7

Strongly Disagree Neutral Strongly Agree

12) I feel a big gap between myself and the other participant.

1 2 3 4 5 6 7

Strongly Disagree Neutral Strongly Agree

**3) STUDENT ROLE**

13) I have a lot in common with other students.

1 2 3 4 5 6 7

Strongly Disagree Neutral Strongly Agree

14) In general, I’m glad to be a student.

1 2 3 4 5 6 7

Strongly Disagree Neutral Strongly Agree

15) Overall, I don’t feel much connectedness with other students.

1 2 3 4 5 6 7

Strongly Disagree Neutral Strongly Agree

16) Other aspects of my life are more important to me than being a student is.

1 2 3 4 5 6 7

Strongly Disagree Neutral Strongly Agree

17) I am usually not very interested to promote or discuss issues important to most students.

1 2 3 4 5 6 7

Strongly Disagree Neutral Strongly Agree

18) I often think about the fact that I’m a student.

1 2 3 4 5 6 7

Strongly Disagree Neutral Strongly Agree

**4) COMPETITION**

19) During the experiment I felt like I should go as fast as I could so that I would be faster than the other person.

1 2 3 4 5 6 7

Strongly Disagree Neutral Strongly Agree

20) I wanted to beat the other participant during the experiment.

1 2 3 4 5 6 7

Strongly Disagree Neutral Strongly Agree

21) I did my absolute best on the computer task in order to win.

1 2 3 4 5 6 7

Strongly Disagree Neutral Strongly Agree

22) It didn’t bother me if the other participant got a better score on the computer task than I did.

1 2 3 4 5 6 7

Strongly Disagree Neutral Strongly Agree

23) Being the most accurate during this experiment was not important to me.

1 2 3 4 5 6 7

Strongly Disagree Neutral Strongly Agree

24) I thought about who would win during the computer task.

1 2 3 4 5 6 7

Strongly Disagree Neutral Strongly Agree
